# Insuffisance aortique syphilitique: à propos d’un cas

**Published:** 2012-07-11

**Authors:** Aimé Arsène Yaméogo, Jean-Baptiste Andonaba, Zakari Nikiéma, Patrice Zabsonré

**Affiliations:** 1 Institut Supérieur des Sciences de la Santé/Université Polytechnique de Bobo-Dioulasso, Burkina Faso; 2 Service de Cardiologie, Centre Hospitalier Universitaire Souro Sanou, Bobo-Dioulasso, Burkina Faso; 3 Unite de Formation et de Recherche-Science De la Santé (UFR-SDS), Université de Ouagadougou, Burkina Faso; 4 Service de cardiologie, Centre Hospitalier Universitaire Yalgado Ouédraogo, Ouagadougou, Burkina Faso

**Keywords:** Insuffisance Aortique, Syphilis tertiaire, Burkina-Faso, Aortic insufficiency, tertiary syphilis, Burkina-Faso

## Abstract

La syphilis tertiaire et ses complications cardiovasculaires sont devenues rares dans les pays développés mais restent encore préoccupante dans nos pays. Les atteintes cardiovasculaires portent fréquemment sur la racine et l’arche aortique. Nous rapportons ici un cas d’insuffisance aortique syphilitique chez un patient de 70 ans admis dans le service de cardiologie du centre hospitalier universitaire de Bobo-Dioulasso. L’examen clinique retrouvait une insuffisance cardiaque globale stade III, un frémissement et un souffle diastolique d’insuffisance aortique importante confirmé à l’échocardiographie Doppler, associés à des douleurs précordiales angineuses. L’examen cutané montrait des lésions à type de gommes syphilitiques à localisations multiples. L’électrocardiogramme objectivait une hypertrophie ventriculaire gauche avec un indice de Sokolov à 49 millimètre et le télécoeur une cardiomégalie avec un index cardio-thoracique à 0,70. La sérologie était positive pour le RPR à 1/8 et le TPHA à 1/640. L’évolution clinique sous la pénicillino-thérapie surveillée et le traitement spécifique de l’insuffisance cardiaque a été favorable. La découverte d’une insuffisance aortique chez les sujets de plus de 60 ans dans nos pays devrait faire rechercher une syphilis tertiaire par une sérologie pour une prise en charge adéquate.

## Introduction

La syphilis tertiaire est devenue très rare avec l’introduction de la pénicillino-thérapie efficace [1]. Ses manifestations cardiovasculaires sont en cours d’élimination dans les pays développés mais constituent toujours une préoccupation dans les pays en voie de développement [2]. La syphilis de par son évolution naturelle peut en effet entrainer à long terme une atteinte de la valve aortique à type de sténose, d’insuffisance ou de coronarite ostiale réalisant l’aortite syphilitique [3]. Nous rapportons ici un cas d’insuffisance aortique sur syphilis tertiaire hospitalisé dans le service de cardiologie du centre hospitalier universitaire de Bobo-Dioulasso.

## Patient et observation

Un homme de 70 ans a été hospitalisé dans notre service le 2 Mars 2012. Il s’agit d’un ancien vigil, de niveau socioéconomique bas, père de six enfants dont trois décédés en bas âge de causes inconnues. Il aurait présenté il y’a environ 30 ans des ulcérations génitales non traitées spontanément guéries. L’interrogatoire retrouvait une toux sèche depuis trois mois, des douleurs rétro-sternales constrictives, une dyspnée stade III de la NYHA et un contexte d’apyrexie. Sur ce tableau s’est greffé des œdèmes des membres inférieurs puis un tableau d’anasarque d’où son hospitalisation en cardiologie pour décompensation cardiaque globale.

L’état général était conservé avec une tachycardie à 115 battements par minute et une polypnée à 27 cycles par minute. La pression artérielle était de 120/50 mmHg.

L’examen physique retrouvait des artères du cou hyper-pulsatiles (signe de Musset) et un frémissement diastolique méso cardiaque. L’auscultation cardiaque mettait en évidence un souffle diastolique latéro-sternale gauche d’insuffisance aortique d’intensité 4/6 et un important éclat du deuxième bruit (B2) au foyer pulmonaire. L’auscultation pulmonaire retrouvait des râles crépitant aux bases pulmonaires.

**Figure 1:**
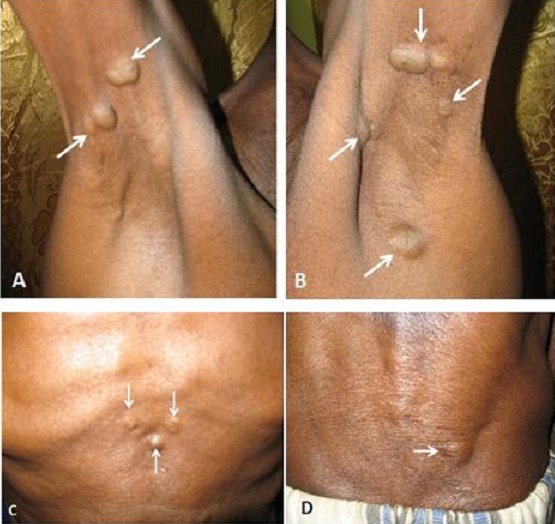
Gommes syphilitiques à localisations multiples (flèches) A: aisselle droite; B: aisselle gauche; C: creux xiphoïdien; D: Région lombaire para vertébrale droite

L’examen cutané montrait des lésions à type de nodules indolores, mobiles à la palpation, profondément enchâssés et de taille variable d’un à deux centimètres de diamètre à localisations multiples (Aisselles, cou, cuir chevelu, creux xiphoïdien et dos), faisant évoquer une gomme syphilitique (Figure 1).

Le bilan para clinique objectivait une sérologie syphilitique positive avec le RPR à 1/8 et le TPHA à 1/640, une fonction rénale normale avec une créatininémie à 89µmol/L et une glycémie normale à 4,1mmol/L. Il n’y avait pas d’hypercholestérolémie.

**Figure 2:**
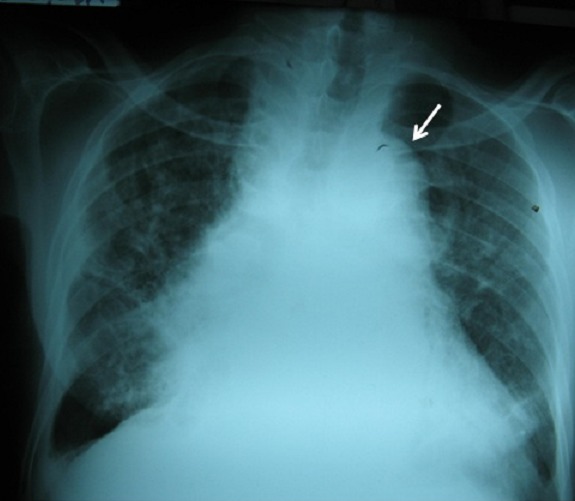
Télécoeur montrant une importante cardiomégalie et une arche aortique déroulée avec élargissement du médiastin moyen (flèche)

Le télécoeur (Figure 2) montrait une cardiomégalie globale (index cardio-thoracique à 0,70), un élargissement du médiastin moyen en rapport avec une dilatation de l’aorte thoracique et des opacités macro-nodulaires plus notables dans le champ pulmonaire droit.

**Figure 3:**
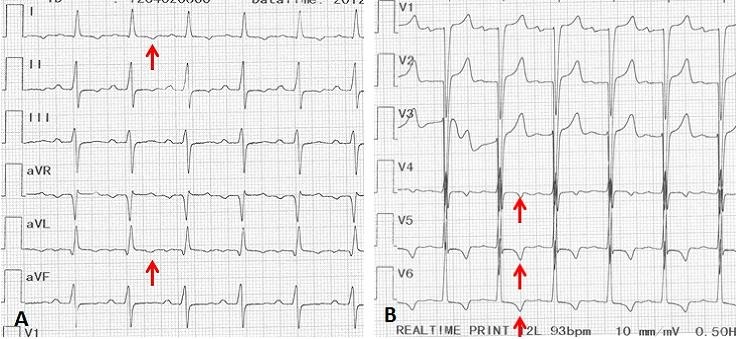
Electrocardiogramme de surface de surface à 12 dérivations (A et B) objectivant une onde T d’ischémie sous épicardique en apico-latéral (flèches rouges)

L’électrocardiogramme de surface (Figure 3) montrait un rythme sinusal régulier, une hypertrophie ventriculaire gauche (Sokolov à 49 mm) et un trouble de la repolarisation à type d’ischémie sous épicardique en apico-latéral.

**Figure 4:**
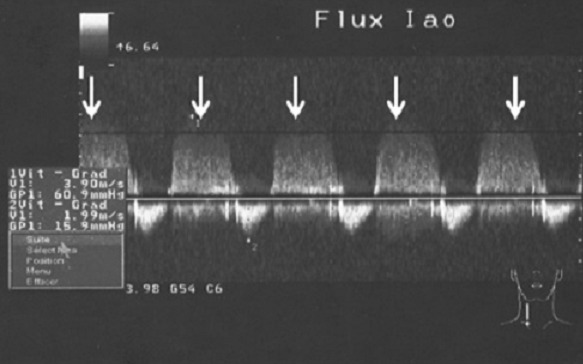
Echographie doppler objectivant un flux d’insuffisance aortique (flèches)

L’échocardiographie Doppler montrait une fraction d’éjection abaissé à 46% par Simpson, une calcification des sigmoïdes aortiques avec l’ouverture inter sigmoïdienne (18mm), sans dilatation de la racine aortique (33mm). L’étude du flux aortique a mis en évidence une insuffisance aortique avec un Vmax à 3,90m/s, un gradient de pression à 61mmHg et un temps de ½ pression= 193ms (Figure 4). Le flux mitral montrait un aspect de trouble de la relaxation et une insuffisance mitrale grade I (Vmax à 1,95m/s, Gradient max à 15,2mmHg). On notait par ailleurs une insuffisance tricuspide (Vmax=2,08m/s). La veine cave inférieure était dilatée. On notait donc une hypertension artérielle pulmonaire avec une pression artérielle pulmonaire systolique estimée à 32,3mmHg.

Un traitement à base de digitalique, de diurétique, de dérivés nitrés ainsi que la Pénicilline G à dose progressive (5UI à 20UI par jour) a été institué. Une corticothérapie par Prednisolone 40mg par jour sous surveillance et respect des règles de la corticothérapie a été administrée par sécurité. L’évolution clinique a été favorable au bout de deux semaines.

## Discussion

Depuis l’introduction de la pénicillino-thérapie, la syphilis tertiaire à travers ses manifestations cardiovasculaires notamment aortiques est devenue une affection très rare dans les pays développés [1]. Si des cas isolés sont toujours rapportés dans les pays en voie de développement [2], la plupart des cas rapportés sont des données d’autopsies [2, 4–6]. Elles mettent la syphilis en troisième position après les causes dégénératives et ischémiques [4, 6]. Comme chez notre patient, il s’agissait d’une affection du sujet de sexe masculin de plus de 60 ans [4, 6, 7]. L’insuffisance aortique non athéromateuse constitue un dysfonctionnement valvulaire fréquent. Elle est rapportée dans la plus part des séries avec des fréquences allant jusqu’à 47% des cas selon les données autopsiques [4, 6]. Cette insuffisance aortique peut s’accompagner d’une insuffisance coronarienne avec manifestation angineuse [8, 9] comme c’était le cas chez notre patient chez qui l’ECG montrait une ischémie sous épicardique. L’absence de coronarographie dans notre contexte n’a pas permis une vérification de l’état des coronaires. Des cas d’insuffisance aortique syphilitique asymptomatique sont cependant rapportés dans la littérature [3].

Des antécédents de maladies sexuellement transmissibles sont souvent retrouvés comme c’est le cas chez notre patient (contact vénérien lointain), mais leurs absence n’exclue pas le diagnostic [7, 8]. Hofmann-Wallenhof [1] avait rapporté que sur environ un tiers des patients qui ne bénéficiaient pas de traitement antibiotique, 10% développeront une complication cardiovasculaire de la syphilis.

Les lésions dermatologiques quand elles existent constituent un élément d’orientation clinique important. Cependant, la sérologie syphilitique positive reste un argument de choix [2]. Selon certains auteurs [2, 5], une insuffisance aortique chez des patients âgés de plus de 50 ans avec ou sans anévrysme de l’aorte qu’elle soit symptomatique ou non, recommande la réalisation d’une sérologie syphilitique.

En se référant au temps de demi-pression, l’IAo de notre patient est importante. Malgré le fait que le traitement médical classique de l’insuffisance cardiaque et la penicillino-thérapie aient apporté une amélioration clinique, un remplacement valvulaire prothétique est à envisager comme le recommande Otani [9] et Guiney [10].

## Conclusion

La syphilis cardiovasculaire est encore une réalité dans nos pays. L’insuffisance aortique en est une manifestation fréquente. Toutes les IAo chez des patients de plus de cinquante ans impliquent la réalisation d’une sérologie syphilitique, un examen cutané minutieux à la recherche d’une syphilis tertiaire pour un traitement adapté.
